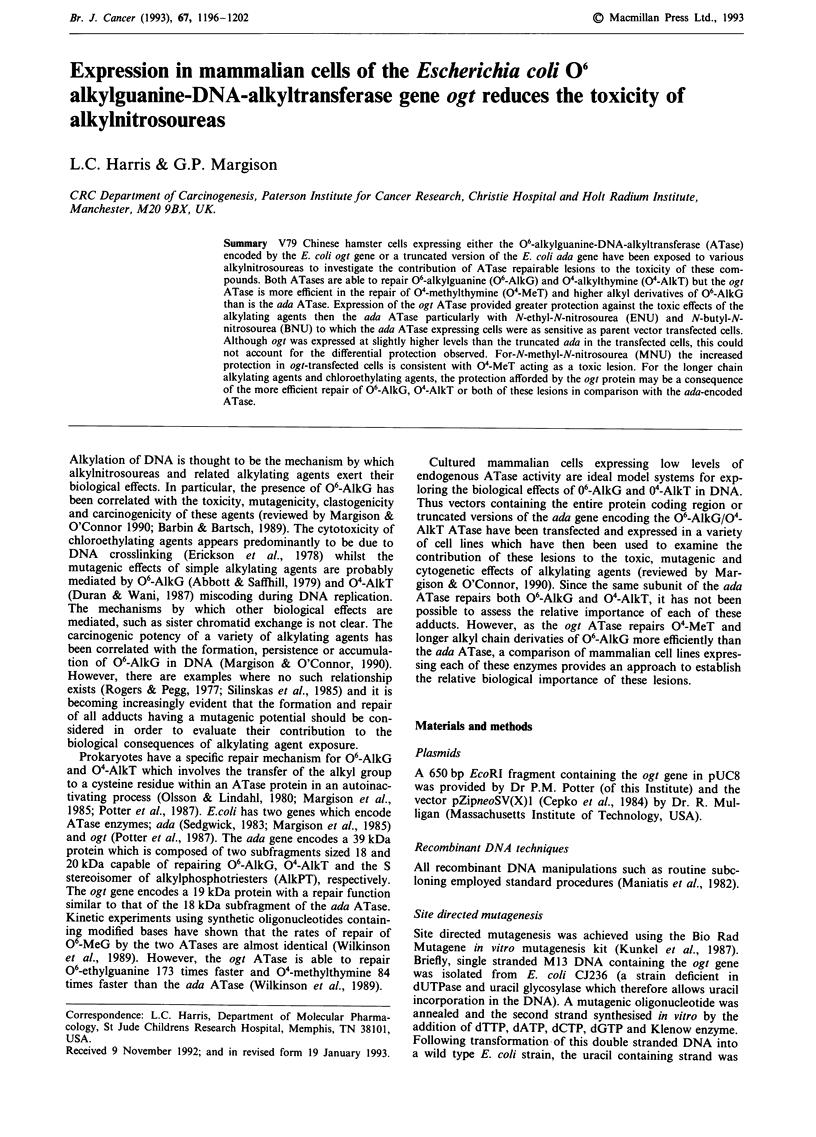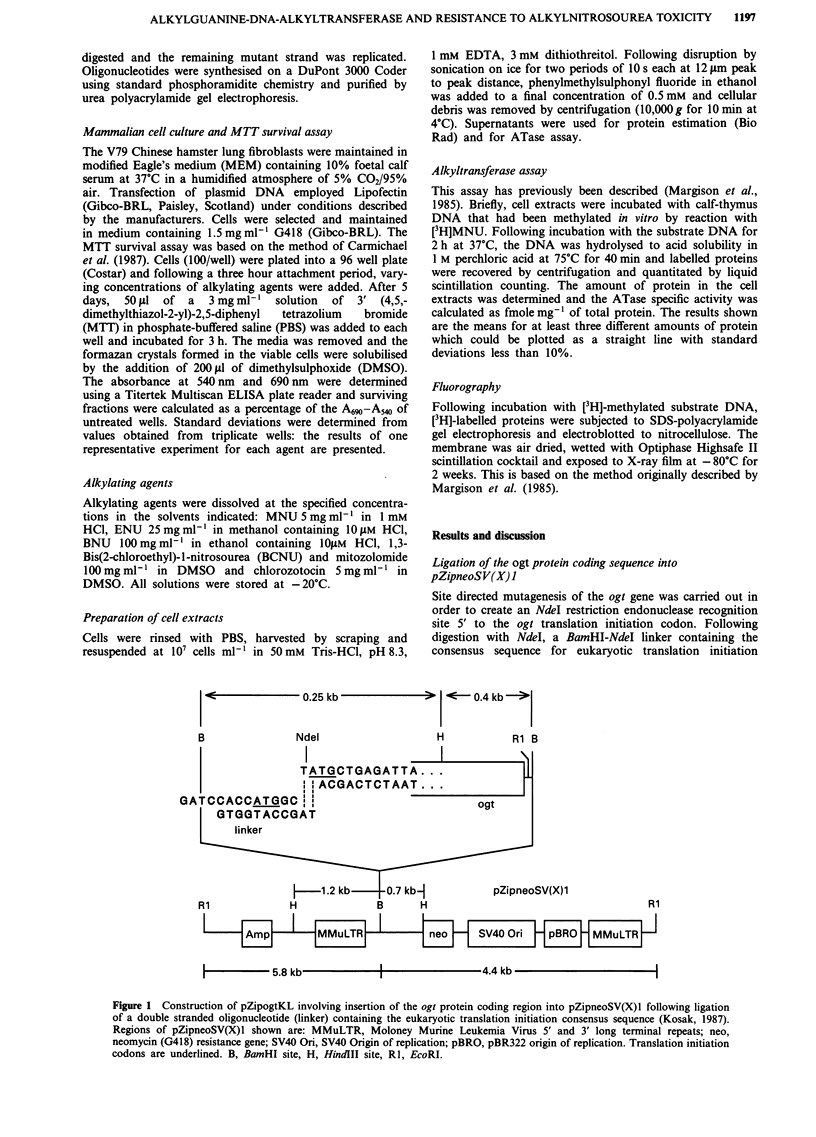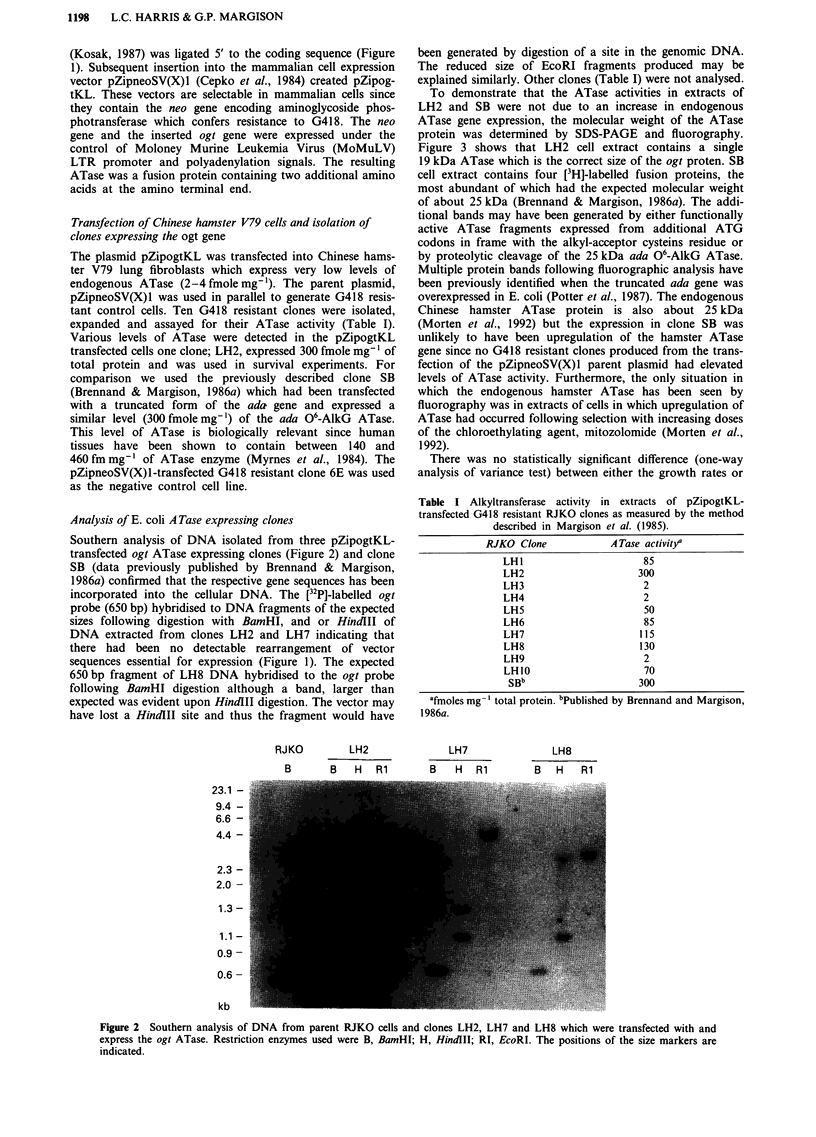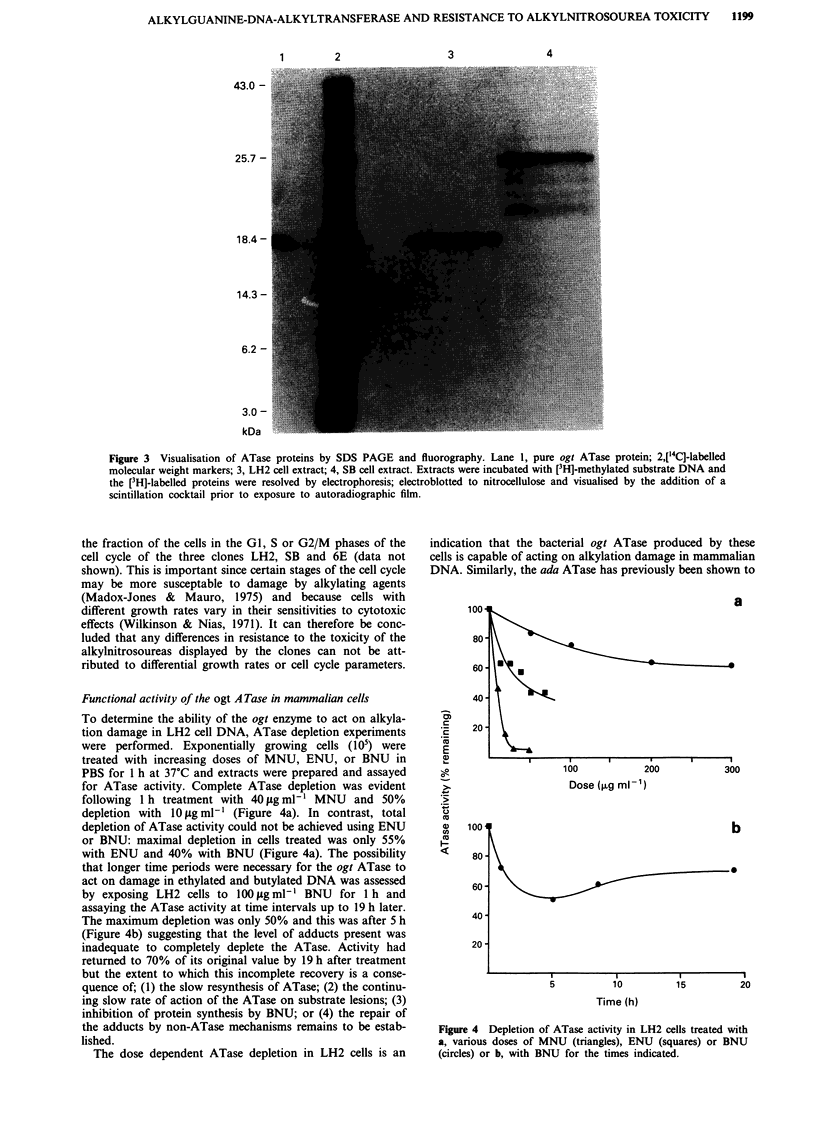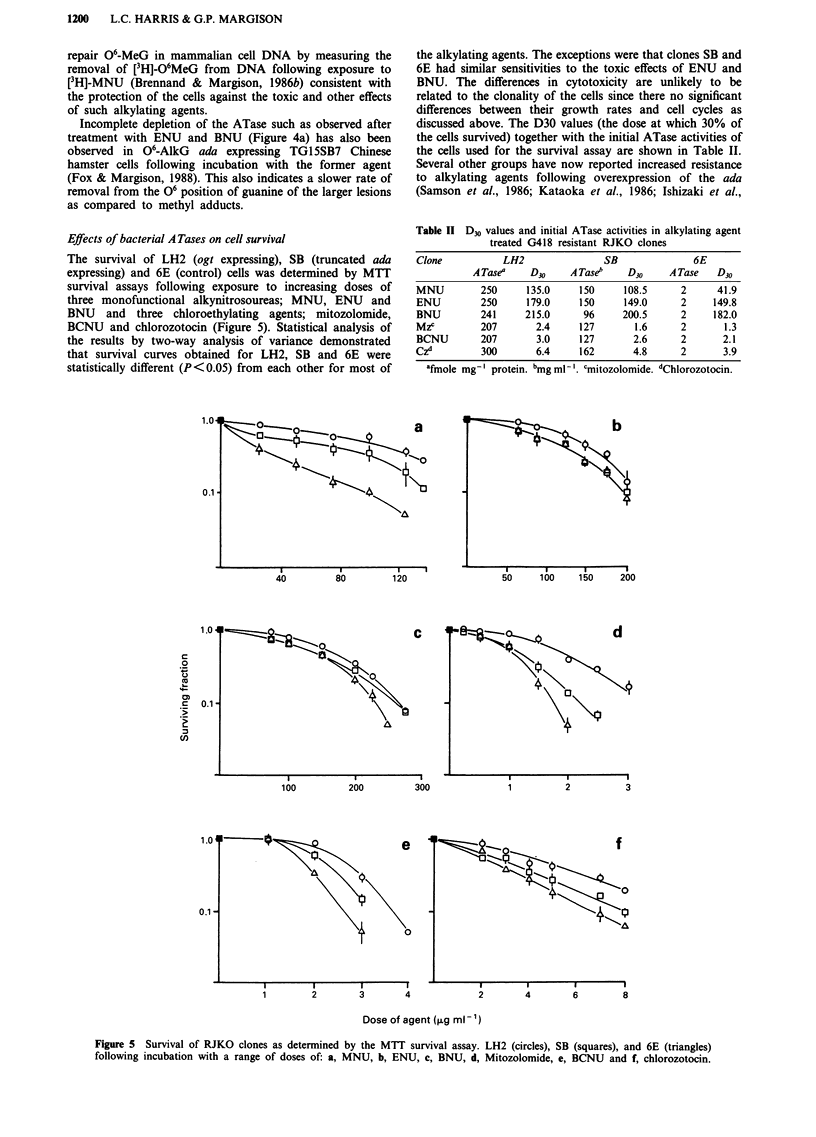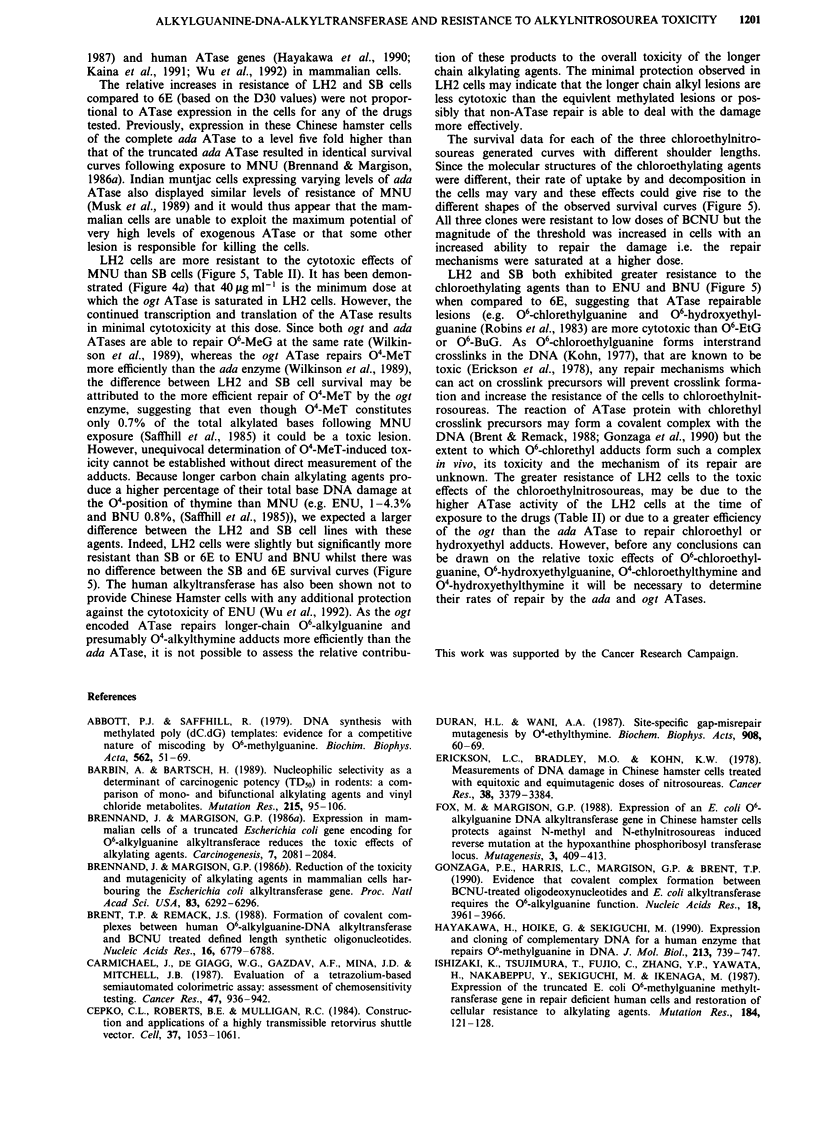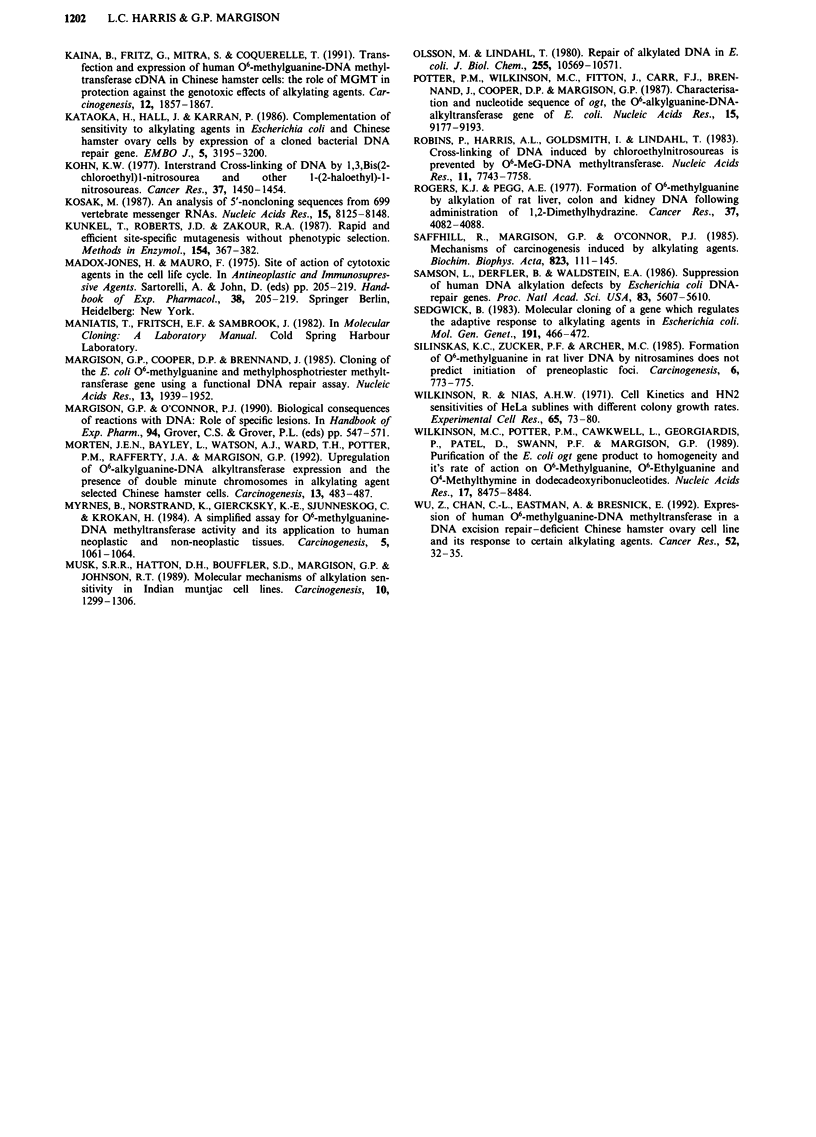# Expression in mammalian cells of the Escherichia coli O6 alkylguanine-DNA-alkyltransferase gene ogt reduces the toxicity of alkylnitrosoureas.

**DOI:** 10.1038/bjc.1993.225

**Published:** 1993-06

**Authors:** L. C. Harris, G. P. Margison

**Affiliations:** CRC Department of Carcinogenesis, Paterson Institute for Cancer Research, Christie Hospital, Manchester, UK.

## Abstract

**Images:**


 © Macmillan Press Ltd., 1993

### Expression in mammalian cells of the *Escherichia coli* O[6] alkylguanine-DNA-alkyltransferase gene *ogt* reduces the toxicity of alkylnitrosoureas

L.C. Harris & G.P. Margison

*CRC Department of Carcinogenesis, Paterson Institute for Cancer Research, Christie Hospital and Holt Radium Institute, Manchester, M20 9BX, UK.*

**Summary** V79 Chinese hamster cells expressing either the O$^6$-alkylguanine-DNA-alkyltransferase (ATase) encoded by the *E. coli ogt* gene or a truncated version of the *E. coli ada* gene have been exposed to various alkylnitrosoureas to investigate the contribution of ATase repairable lesions to the toxicity of these compounds. Both ATases are able to repair O$^6$-alkylguanine (O$^6$-AlkG) and O$^4$-alkylthymine (O$^4$-AlkT) but the *ogt* ATase is more efficient in the repair of O$^4$-methylthymine (O$^4$-MeT) and higher alkyl derivatives of O$^6$-AlkG than is the *ada* ATase. Expression of the *ogt* ATase provided greater protection against the toxic effects of the alkylating agents then the *ada* ATase particularly with *N*-ethyl-*N*-nitrosourea (ENU) and *N*-butyl-*N*-nitrosourea (BNU) to which the *ada* ATase expressing cells were as sensitive as parent vector transfected cells. Although *ogt* was expressed at slightly higher levels than the truncated *ada* in the transfected cells, this could not account for the differential protection observed. For-*N*-methyl-*N*-nitrosourea (MNU) the increased protection in *ogt*-transfected cells is consistent with O$^4$-MeT acting as a toxic lesion. For the longer chain alkylating agents and chloroethylating agents, the protection afforded by the *ogt* protein may be a consequence of the more efficient repair of O$^6$-AlkG, O$^4$-AlkT or both of these lesions in comparison with the *ada*-encoded ATase.

Alkylation of DNA is thought to be the mechanism by which alkylnitrosoureas and related alkylating agents exert their biological effects. In particular, the presence of O$^6$-AlkG has been correlated with the toxicity, mutagenicity, clastogenicity and carcinogenicity of these agents (reviewed by Margison & O'Connor 1990; Barbin & Bartsch, 1989). The cytotoxicity of chloroethylating agents appears predominantly to be due to DNA crosslinking (Erickson *et al.*, 1978) whilst the mutagenic effects of simple alkylating agents are probably mediated by O$^6$-AlkG (Abbott & Saffhill, 1979) and O$^4$-AlkT (Duran & Wani, 1987) miscoding during DNA replication. The mechanisms by which other biological effects are mediated, such as sister chromatid exchange is not clear. The carcinogenic potency of a variety of alkylating agents has been correlated with the formation, persistence or accumulation of O$^6$-AlkG in DNA (Margison & O'Connor, 1990). However, there are examples where no such relationship exists (Rogers & Pegg, 1977; Silinskas *et al.*, 1985) and it is becoming increasingly evident that the formation and repair of all adducts having a mutagenic potential should be considered in order to evaluate their contribution to the biological consequences of alkylating agent exposure.

Prokaryotes have a specific repair mechanism for O$^6$-AlkG and O$^4$-AlkT which involves the transfer of the alkyl group to a cysteine residue within an ATase protein in an autoinactivating process (Olsson & Lindahl, 1980; Margison *et al.*, 1985; Potter *et al.*, 1987). *E.coli* has two genes which encode ATase enzymes; *ada* (Sedgwick, 1983; Margison *et al.*, 1985) and *ogt* (Potter *et al.*, 1987). The *ada* gene encodes a 39 kDa protein which is composed of two subfragments sized 18 and 20 kDa capable of repairing O$^6$-AlkG, O$^4$-AlkT and the S stereoisomer of alkylphosphotriesters (AlkPT), respectively. The *ogt* gene encodes a 19 kDa protein with a repair function similar to that of the 18 kDa subfragment of the *ada* ATase. Kinetic experiments using synthetic oligonucleotides containing modified bases have shown that the rates of repair of O$^6$-MeG by the two ATases are almost identical (Wilkinson *et al.*, 1989). However, the *ogt* ATase is able to repair O$^6$-ethylguanine 173 times faster and O$^4$-methylthymine 84 times faster than the *ada* ATase (Wilkinson *et al.*, 1989).

Correspondence: L.C. Harris, Department of Molecular Pharmacology, St Jude Childrens Research Hospital, Memphis, TN 38101, USA.
Received 9 November 1992; and in revised form 19 January 1993.

Cultured mammalian cells expressing low levels of endogenous ATase activity are ideal model systems for exploring the biological effects of 0$^6$-AlkG and 0$^4$-AlkT in DNA. Thus vectors containing the entire protein coding region or truncated versions of the *ada* gene encoding the O$^6$-AlkG/O$^4$-AlkT ATase have been transfected and expressed in a variety of cell lines which have then been used to examine the contribution of these lesions to the toxic, mutagenic and cytogenetic effects of alkylating agents (reviewed by Margison & O'Connor, 1990). Since the same subunit of the *ada* ATase repairs both O$^6$-AlkG and O$^4$-AlkT, it has not been possible to assess the relative importance of each of these adducts. However, as the *ogt* ATase repairs O$^4$-MeT and longer alkyl chain derivaties of O$^6$-AlkG more efficiently than the *ada* ATase, a comparison of mammalian cell lines expressing each of these enzymes provides an approach to establish the relative biological importance of these lesions.

### Materials and methods

### *Plasmids*

A 650 bp *Eco*RI fragment containing the *ogt* gene in pUC8 was provided by Dr P.M. Potter (of this Institute) and the vector pZip*neo*SV(X)1 (Cepko *et al.*, 1984) by Dr. R. Mulligan (Massachusetts Institute of Technology, USA).

### *Recombinant DNA techniques*

All recombinant DNA manipulations such as routine subcloning employed standard procedures (Maniatis *et al.*, 1982).

### *Site directed mutagenesis*

Site directed mutagenesis was achieved using the Bio Rad Mutagene *in vitro* mutagenesis kit (Kunkel *et al.*, 1987). Briefly, single stranded M13 DNA containing the *ogt* gene was isolated from *E. coli* CJ236 (a strain deficient in dUTPase and uracil glycosylase which therefore allows uracil incorporation in the DNA). A mutagenic oligonucleotide was annealed and the second strand synthesised *in vitro* by the addition of dTTP, dATP, dCTP, dGTP and Klenow enzyme. Following transformation of this double stranded DNA into a wild type *E. coli* strain, the uracil containing strand was digested and the remaining mutant strand was replicated. Oligonucleotides were synthesised on a DuPont 3000 Coder using standard phosphoramidite chemistry and purified by urea polyacrylamide gel electrophoresis.

*Mammalian cell culture and MTT survival assay*

The V79 Chinese hamster lung fibroblasts were maintained in modified Eagle's medium (MEM) containing 10% foetal calf serum at 37°C in a humidified atmosphere of 5% $CO_2$/95% air. Transfection of plasmid DNA employed Lipofectin (Gibco-BRL, Paisley, Scotland) under conditions described by the manufacturers. Cells were selected and maintained in medium containing 1.5 mg ml$^{-1}$ G418 (Gibco-BRL). The MTT survival assay was based on the method of Carmichael *et al.* (1987). Cells (100/well) were plated into a 96 well plate (Costar) and following a three hour attachment period, varying concentrations of alkylating agents were added. After 5 days, 50 µl of a 3 mg ml$^{-1}$ solution of 3′ (4,5,-dimethylthiazol-2-yl)-2,5-diphenyl tetrazolium bromide (MTT) in phosphate-buffered saline (PBS) was added to each well and incubated for 3 h. The media was removed and the formazan crystals formed in the viable cells were solubilised by the addition of 200 µl of dimethylsulphoxide (DMSO). The absorbance at 540 nm and 690 nm were determined using a Titertek Multiscan ELISA plate reader and surviving fractions were calculated as a percentage of the $A_{690}-A_{540}$ of untreated wells. Standard deviations were determined from values obtained from triplicate wells: the results of one representative experiment for each agent are presented.

*Alkylating agents*

Alkylating agents were dissolved at the specified concentrations in the solvents indicated: MNU 5 mg ml$^{-1}$ in 1 mM HCl, ENU 25 mg ml$^{-1}$ in methanol containing 10 µM HCl, BNU 100 mg ml$^{-1}$ in ethanol containing 10µM HCl, 1,3-Bis(2-chloroethyl)-1-nitrosourea (BCNU) and mitozolomide 100 mg ml$^{-1}$ in DMSO and chlorozotocin 5 mg ml$^{-1}$ in DMSO. All solutions were stored at −20°C.

*Preparation of cell extracts*

Cells were rinsed with PBS, harvested by scraping and resuspended at $10^7$ cells ml$^{-1}$ in 50 mM Tris-HCl, pH 8.3, 1 mM EDTA, 3 mM dithiothreitol. Following disruption by sonication on ice for two periods of 10 s each at 12 µm peak to peak distance, phenylmethylsulphonyl fluoride in ethanol was added to a final concentration of 0.5 mM and cellular debris was removed by centrifugation (10,000 *g* for 10 min at 4°C). Supernatants were used for protein estimation (Bio Rad) and for ATase assay.

*Alkyltransferase assay*

This assay has previously been described (Margison *et al.*, 1985). Briefly, cell extracts were incubated with calf-thymus DNA that had been methylated *in vitro* by reaction with [$^3$H]MNU. Following incubation with the substrate DNA for 2 h at 37°C, the DNA was hydrolysed to acid solubility in 1 M perchloric acid at 75°C for 40 min and labelled proteins were recovered by centrifugation and quantitated by liquid scintillation counting. The amount of protein in the cell extracts was determined and the ATase specific activity was calculated as fmole mg$^{-1}$ of total protein. The results shown are the means for at least three different amounts of protein which could be plotted as a straight line with standard deviations less than 10%.

*Fluorography*

Following incubation with [$^3$H]-methylated substrate DNA, [$^3$H]-labelled proteins were subjected to SDS-polyacrylamide gel electrophoresis and electroblotted to nitrocellulose. The membrane was air dried, wetted with Optiphase Highsafe II scintillation cocktail and exposed to X-ray film at −80°C for 2 weeks. This is based on the method originally described by Margison *et al.* (1985).

### Results and discussion

*Ligation of the* ogt *protein coding sequence into pZipneoSV(X)1*

Site directed mutagenesis of the *ogt* gene was carried out in order to create an *Nde*I restriction endonuclease recognition site 5′ to the *ogt* translation initiation codon. Following digestion with *Nde*I, a *Bam*HI-*Nde*I linker containing the consensus sequence for eukaryotic translation initiation

**Figure 1** Construction of pZipogtKL involving insertion of the *ogt* protein coding region into pZipneoSV(X)1 following ligation of a double stranded oligonucleotide (linker) containing the eukaryotic translation initiation consensus sequence (Kosak, 1987). Regions of pZipneoSV(X)1 shown are: MMuLTR, Moloney Murine Leukemia Virus 5′ and 3′ long terminal repeats; neo, neomycin (G418) resistance gene; SV40 Ori, SV40 Origin of replication; pBRO, pBR322 origin of replication. Translation initiation codons are underlined. B, *Bam*HI site, H, *Hind*III site, R1, *Eco*RI.

(Kosak, 1987) was ligated 5′ to the coding sequence (Figure 1). Subsequent insertion into the mammalian cell expression vector pZipneoSV(X)1 (Cepko *et al.*, 1984) created pZipogtKL. These vectors are selectable in mammalian cells since they contain the *neo* gene encoding aminoglycoside phosphotransferase which confers resistance to G418. The *neo* gene and the inserted *ogt* gene were expressed under the control of Moloney Murine Leukemia Virus (MoMuLV) LTR promoter and polyadenylation signals. The resulting ATase was a fusion protein containing two additional amino acids at the amino terminal end.

*Transfection of Chinese hamster V79 cells and isolation of clones expressing the* ogt *gene*

The plasmid pZipogtKL was transfected into Chinese hamster V79 lung fibroblasts which express very low levels of endogenous ATase (2–4 fmole $mg^{-1}$). The parent plasmid, pZipneoSV(X)1 was used in parallel to generate G418 resistant control cells. Ten G418 resistant clones were isolated, expanded and assayed for their ATase activity (Table I). Various levels of ATase were detected in the pZipogtKL transfected cells one clone; LH2, expressed 300 fmole $mg^{-1}$ of total protein and was used in survival experiments. For comparison we used the previously described clone SB (Brennand & Margison, 1986*a*) which had been transfected with a truncated form of the *ada* gene and expressed a similar level (300 fmole $mg^{-1}$) of the *ada* $O^6$-AlkG ATase. This level of ATase is biologically relevant since human tissues have been shown to contain between 140 and 460 fm $mg^{-1}$ of ATase enzyme (Myrnes *et al.*, 1984). The pZipneoSV(X)1-transfected G418 resistant clone 6E was used as the negative control cell line.

*Analysis of* E. coli *ATase expressing clones*

Southern analysis of DNA isolated from three pZipogtKL-transfected *ogt* ATase expressing clones (Figure 2) and clone SB (data previously published by Brennand & Margison, 1986*a*) confirmed that the respective gene sequences has been incorporated into the cellular DNA. The [$^{32}P$]-labelled *ogt* probe (650 bp) hybridised to DNA fragments of the expected sizes following digestion with *Bam*HI, and or *Hind*III of DNA extracted from clones LH2 and LH7 indicating that there had been no detectable rearrangement of vector sequences essential for expression (Figure 1). The expected 650 bp fragment of LH8 DNA hybridised to the *ogt* probe following *Bam*HI digestion although a band, larger than expected was evident upon *Hind*III digestion. The vector may have lost a *Hind*III site and thus the fragment would have been generated by digestion of a site in the genomic DNA. The reduced size of EcoRI fragments produced may be explained similarly. Other clones (Table I) were not analysed.

To demonstrate that the ATase activities in extracts of LH2 and SB were not due to an increase in endogenous ATase gene expression, the molecular weight of the ATase protein was determined by SDS-PAGE and fluorography. Figure 3 shows that LH2 cell extract contains a single 19 kDa ATase which is the correct size of the *ogt* proten. SB cell extract contains four [$^3H$]-labelled fusion proteins, the most abundant of which had the expected molecular weight of about 25 kDa (Brennand & Margison, 1986*a*). The additional bands may have been generated by either functionally active ATase fragments expressed from additional ATG codons in frame with the alkyl-acceptor cysteins residue or by proteolytic cleavage of the 25 kDa *ada* $O^6$-AlkG ATase. Multiple protein bands following fluorographic analysis have been previously identified when the truncated *ada* gene was overexpressed in E. coli (Potter *et al.*, 1987). The endogenous Chinese hamster ATase protein is also about 25 kDa (Morten *et al.*, 1992) but the expression in clone SB was unlikely to have been upregulation of the hamster ATase gene since no G418 resistant clones produced from the transfection of the pZipneoSV(X)1 parent plasmid had elevated levels of ATase activity. Furthermore, the only situation in which the endogenous hamster ATase has been seen by fluorography was in extracts of cells in which upregulation of ATase had occurred following selection with increasing doses of the chloroethylating agent, mitozolomide (Morten *et al.*, 1992).

There was no statistically significant difference (one-way analysis of variance test) between either the growth rates or

**Table I** Alkyltransferase activity in extracts of pZipogtKL-transfected G418 resistant RJKO clones as measured by the method described in Margison *et al.* (1985).

| *RJKO Clone* | *ATase activity*[a] |
|---|---|
| LH1 | 85 |
| LH2 | 300 |
| LH3 | 2 |
| LH4 | 2 |
| LH5 | 50 |
| LH6 | 85 |
| LH7 | 115 |
| LH8 | 130 |
| LH9 | 2 |
| LH10 | 70 |
| SB[b] | 300 |

[a]fmoles $mg^{-1}$ total protein. [b]Published by Brennand and Margison, 1986*a*.

**Figure 2** Southern analysis of DNA from parent RJKO cells and clones LH2, LH7 and LH8 which were transfected with and express the *ogt* ATase. Restriction enzymes used were B, *Bam*HI; H, *Hind*III; RI, *Eco*RI. The positions of the size markers are indicated.

**Figure 3** Visualisation of ATase proteins by SDS PAGE and fluorography. Lane 1, pure *ogt* ATase protein; 2,[$^{14}$C]-labelled molecular weight markers; 3, LH2 cell extract; 4, SB cell extract. Extracts were incubated with [$^{3}$H]-methylated substrate DNA and the [$^{3}$H]-labelled proteins were resolved by electrophoresis; electroblotted to nitrocellulose and visualised by the addition of a scintillation cocktail prior to exposure to autoradiographic film.

the fraction of the cells in the G1, S or G2/M phases of the cell cycle of the three clones LH2, SB and 6E (data not shown). This is important since certain stages of the cell cycle may be more susceptable to damage by alkylating agents (Madox-Jones & Mauro, 1975) and because cells with different growth rates vary in their sensitivities to cytotoxic effects (Wilkinson & Nias, 1971). It can therefore be concluded that any differences in resistance to the toxicity of the alkylnitrosoureas displayed by the clones can not be attributed to differential growth rates or cell cycle parameters.

### *Functional activity of the* ogt *ATase in mammalian cells*

To determine the ability of the *ogt* enzyme to act on alkylation damage in LH2 cell DNA, ATase depletion experiments were performed. Exponentially growing cells ($10^5$) were treated with increasing doses of MNU, ENU, or BNU in PBS for 1 h at 37°C and extracts were prepared and assayed for ATase activity. Complete ATase depletion was evident following 1 h treatment with 40 $\mu$g ml$^{-1}$ MNU and 50% depletion with 10 $\mu$g ml$^{-1}$ (Figure 4a). In contrast, total depletion of ATase activity could not be achieved using ENU or BNU: maximal depletion in cells treated was only 55% with ENU and 40% with BNU (Figure 4a). The possibility that longer time periods were necessary for the *ogt* ATase to act on damage in ethylated and butylated DNA was assessed by exposing LH2 cells to 100 $\mu$g ml$^{-1}$ BNU for 1 h and assaying the ATase activity at time intervals up to 19 h later. The maximum depletion was only 50% and this was after 5 h (Figure 4b) suggesting that the level of adducts present was inadequate to completely deplete the ATase. Activity had returned to 70% of its original value by 19 h after treatment but the extent to which this incomplete recovery is a consequence of; (1) the slow resynthesis of ATase; (2) the continuing slow rate of action of the ATase on substrate lesions; (3) inhibition of protein synthesis by BNU; or (4) the repair of the adducts by non-ATase mechanisms remains to be established.

The dose dependent ATase depletion in LH2 cells is an indication that the bacterial *ogt* ATase produced by these cells is capable of acting on alkylation damage in mammalian DNA. Similarly, the *ada* ATase has previously been shown to

**Figure 4** Depletion of ATase activity in LH2 cells treated with **a**, various doses of MNU (triangles), ENU (squares) or BNU (circles) or **b**, with BNU for the times indicated.

repair $O^6$-MeG in mammalian cell DNA by measuring the removal of [$^3$H]-$O^6$MeG from DNA following exposure to [$^3$H]-MNU (Brennand & Margison, 1986*b*) consistent with the protection of the cells against the toxic and other effects of such alkylating agents.

Incomplete depletion of the ATase such as observed after treatment with ENU and BNU (Figure 4a) has also been observed in $O^6$-AlkG *ada* expressing TG15SB7 Chinese hamster cells following incubation with the former agent (Fox & Margison, 1988). This also indicates a slower rate of removal from the $O^6$ position of guanine of the larger lesions as compared to methyl adducts.

*Effects of bacterial ATases on cell survival*

The survival of LH2 (*ogt* expressing), SB (truncated *ada* expressing) and 6E (control) cells was determined by MTT survival assays following exposure to increasing doses of three monofunctional alkynitrosoureas; MNU, ENU and BNU and three chloroethylating agents; mitozolomide, BCNU and chlorozotocin (Figure 5). Statistical analysis of the results by two-way analysis of variance demonstrated that survival curves obtained for LH2, SB and 6E were statistically different ($P<0.05$) from each other for most of the alkylating agents. The exceptions were that clones SB and 6E had similar sensitivities to the toxic effects of ENU and BNU. The differences in cytotoxicity are unlikely to be related to the clonality of the cells since there no significant differences between their growth rates and cell cycles as discussed above. The D30 values (the dose at which 30% of the cells survived) together with the initial ATase activities of the cells used for the survival assay are shown in Table II. Several other groups have now reported increased resistance to alkylating agents following overexpression of the *ada* (Samson *et al.*, 1986; Kataoka *et al.*, 1986; Ishizaki *et al.*,

**Table II** $D_{30}$ values and initial ATase activities in alkylating agent treated G418 resistant RJKO clones

| *Clone* | *LH2* | | *SB* | | *6E* | |
|---|---|---|---|---|---|---|
| | *ATase*[a] | $D_{30}$ | *ATase*[b] | $D_{30}$ | *ATase* | $D_{30}$ |
| MNU | 250 | 135.0 | 150 | 108.5 | 2 | 41.9 |
| ENU | 250 | 179.0 | 150 | 149.0 | 2 | 149.8 |
| BNU | 241 | 215.0 | 96 | 200.5 | 2 | 182.0 |
| Mz[c] | 207 | 2.4 | 127 | 1.6 | 2 | 1.3 |
| BCNU | 207 | 3.0 | 127 | 2.6 | 2 | 2.1 |
| Cz[d] | 300 | 6.4 | 162 | 4.8 | 2 | 3.9 |

[a]fmole $mg^{-1}$ protein. [b]$mg\ ml^{-1}$. [c]mitozolomide. [d]Chlorozotocin.

**Figure 5** Survival of RJKO clones as determined by the MTT survival assay. LH2 (circles), SB (squares), and 6E (triangles) following incubation with a range of doses of: **a**, MNU, **b**, ENU, **c**, BNU, **d**, Mitozolomide, **e**, BCNU and **f**, chlorozotocin.

1987) and human ATase genes (Hayakawa *et al.*, 1990; Kaina *et al.*, 1991; Wu *et al.*, 1992) in mammalian cells.

The relative increases in resistance of LH2 and SB cells compared to 6E (based on the D30 values) were not proportional to ATase expression in the cells for any of the drugs tested. Previously, expression in these Chinese hamster cells of the complete *ada* ATase to a level five fold higher than that of the truncated *ada* ATase resulted in identical survival curves following exposure to MNU (Brennand & Margison, 1986*a*). Indian muntjac cells expressing varying levels of *ada* ATase also displayed similar levels of resistance of MNU (Musk *et al.*, 1989) and it would thus appear that the mammalian cells are unable to exploit the maximum potential of very high levels of exogenous ATase or that some other lesion is responsible for killing the cells.

LH2 cells are more resistant to the cytotoxic effects of MNU than SB cells (Figure 5, Table II). It has been demonstrated (Figure 4*a*) that 40 $\mu g\ ml^{-1}$ is the minimum dose at which the *ogt* ATase is saturated in LH2 cells. However, the continued transcription and translation of the ATase results in minimal cytotoxicity at this dose. Since both *ogt* and *ada* ATases are able to repair $O^6$-MeG at the same rate (Wilkinson *et al.*, 1989), whereas the *ogt* ATase repairs $O^4$-MeT more efficiently than the *ada* enzyme (Wilkinson *et al.*, 1989), the difference between LH2 and SB cell survival may be attributed to the more efficient repair of $O^4$-MeT by the *ogt* enzyme, suggesting that even though $O^4$-MeT constitutes only 0.7% of the total alkylated bases following MNU exposure (Saffhill *et al.*, 1985) it could be a toxic lesion. However, unequivocal determination of $O^4$-MeT-induced toxicity cannot be established without direct measurement of the adducts. Because longer carbon chain alkylating agents produce a higher percentage of their total base DNA damage at the $O^4$-position of thymine than MNU (e.g. ENU, 1–4.3% and BNU 0.8%, (Saffhill *et al.*, 1985)), we expected a larger difference between the LH2 and SB cell lines with these agents. Indeed, LH2 cells were slightly but significantly more resistant than SB or 6E to ENU and BNU whilst there was no difference between the SB and 6E survival curves (Figure 5). The human alkyltransferase has also been shown not to provide Chinese Hamster cells with any additional protection against the cytotoxicity of ENU (Wu *et al.*, 1992). As the *ogt* encoded ATase repairs longer-chain $O^6$-alkylguanine and presumably $O^4$-alkylthymine adducts more efficiently than the *ada* ATase, it is not possible to assess the relative contribution of these products to the overall toxicity of the longer chain alkylating agents. The minimal protection observed in LH2 cells may indicate that the longer chain alkyl lesions are less cytotoxic than the equivlent methylated lesions or possibly that non-ATase repair is able to deal with the damage more effectively.

The survival data for each of the three chloroethylnitrosoureas generated curves with different shoulder lengths. Since the molecular structures of the chloroethylating agents were different, their rate of uptake by and decomposition in the cells may vary and these effects could give rise to the different shapes of the observed survival curves (Figure 5). All three clones were resistant to low doses of BCNU but the magnitude of the threshold was increased in cells with an increased ability to repair the damage i.e. the repair mechanisms were saturated at a higher dose.

LH2 and SB both exhibited greater resistance to the chloroethylating agents than to ENU and BNU (Figure 5) when compared to 6E, suggesting that ATase repairable lesions (e.g. $O^6$-chlorethylguanine and $O^6$-hydroxyethylguanine (Robins *et al.*, 1983) are more cytotoxic than $O^6$-EtG or $O^6$-BuG. As $O^6$-chloroethylguanine forms interstrand crosslinks in the DNA (Kohn, 1977), that are known to be toxic (Erickson *et al.*, 1978), any repair mechanisms which can act on crosslink precursors will prevent crosslink formation and increase the resistance of the cells to chloroethylnitrosoureas. The reaction of ATase protein with chlorethyl crosslink precursors may form a covalent complex with the DNA (Brent & Remack, 1988; Gonzaga *et al.*, 1990) but the extent to which $O^6$-chlorethyl adducts form such a complex *in vivo*, its toxicity and the mechanism of its repair are unknown. The greater resistance of LH2 cells to the toxic effects of the chloroethylnitrosoureas, may be due to the higher ATase activity of the LH2 cells at the time of exposure to the drugs (Table II) or due to a greater efficiency of the *ogt* than the *ada* ATase to repair chloroethyl or hydroxyethyl adducts. However, before any conclusions can be drawn on the relative toxic effects of $O^6$-chloroethylguanine, $O^6$-hydroxyethylguanine, $O^4$-chloroethylthymine and $O^4$-hydroxyethylthymine it will be necessary to determine their rates of repair by the *ada* and *ogt* ATases.

This work was supported by the Cancer Research Campaign.

### References

ABBOTT, P.J. & SAFFHILL, R. (1979). DNA synthesis with methylated poly (dC.dG) templates: evidence for a competitive nature of miscoding by $O^6$-methylguanine. *Biochim. Biophys. Acta*, **562,** 51–69.

BARBIN, A. & BARTSCH, H. (1989). Nucleophilic selectivity as a determinant of carcinogenic potency ($TD_{50}$) in rodents: a comparison of mono- and bifunctional alkylating agents and vinyl chloride metabolites. *Mutation Res.*, **215,** 95–106.

BRENNAND, J. & MARGISON, G.P. (1986*a*). Expression in mammalian cells of a truncated *Escherichia coli* gene encoding for $O^6$-alkylguanine alkyltransferace reduces the toxic effects of alkylating agents. *Carcinogenesis*, **7,** 2081–2084.

BRENNAND, J. & MARGISON, G.P. (1986*b*). Reduction of the toxicity and mutagenicity of alkylating agents in mammalian cells harbouring the *Escherichia coli* alkyltransferase gene. *Proc. Natl Acad Sci. USA*, **83,** 6292–6296.

BRENT, T.P. & REMACK, J.S. (1988). Formation of covalent complexes between human $O^6$-alkylguanine-DNA alkyltransferase and BCNU treated defined length synthetic oligonucleotides. *Nucleic Acids Res.*, **16,** 6779–6788.

CARMICHAEL, J., DE GIAGG, W.G., GAZDAV, A.F., MINA, J.D. & MITCHELL, J.B. (1987). Evaluation of a tetrazolium-based semiautomated colorimetric assay: assessment of chemosensitivity testing. *Cancer Res.*, **47,** 936–942.

CEPKO, C.L., ROBERTS, B.E. & MULLIGAN, R.C. (1984). Construction and applications of a highly transmissible retorvirus shuttle vector. *Cell*, **37,** 1053–1061.

DURAN, H.L. & WANI, A.A. (1987). Site-specific gap-misrepair mutagenesis by $O^4$-ethylthymine. *Biochem. Biophys. Acts*, **908,** 60–69.

ERICKSON, L.C., BRADLEY, M.O. & KOHN, K.W. (1978). Measurements of DNA damage in Chinese hamster cells treated with equitoxic and equimutagenic doses of nitrosoureas. *Cancer Res.*, **38,** 3379–3384.

FOX, M. & MARGISON, G.P. (1988). Expression of an *E. coli* $O^6$-alkylguanine DNA alkyltransferase gene in Chinese hamster cells protects against N-methyl and N-ethylnitrosoureas induced reverse mutation at the hypoxanthine phosphoribosyl transferase locus. *Mutagenesis*, **3,** 409–413.

GONZAGA, P.E., HARRIS, L.C., MARGISON, G.P. & BRENT, T.P. (1990). Evidence that covalent complex formation between BCNU-treated oligodeoxynucleotides and *E. coli* alkyltransferase requires the $O^6$-alkylguanine function. *Nucleic Acids Res.*, **18,** 3961–3966.

HAYAKAWA, H., HOIKE, G. & SEKIGUCHI, M. (1990). Expression and cloning of complementary DNA for a human enzyme that repairs $O^6$-methylguanine in DNA. *J. Mol. Biol.*, **213,** 739–747.

ISHIZAKI, K., TSUJIMURA, T., FUJIO, C., ZHANG, Y.P., YAWATA, H., NAKABEPPU, Y., SEKIGUCHI, M. & IKENAGA, M. (1987). Expression of the truncated E. coli $O^6$-methylguanine methyltransferase gene in repair deficient human cells and restoration of cellular resistance to alkylating agents. *Mutation Res.*, **184,** 121–128.

KAINA, B., FRITZ, G., MITRA, S. & COQUERELLE, T. (1991). Transfection and expression of human $O^6$-methylguanine-DNA methyltransferase cDNA in Chinese hamster cells: the role of MGMT in protection against the genotoxic effects of alkylating agents. *Carcinogenesis*, **12**, 1857-1867.

KATAOKA, H., HALL, J. & KARRAN, P. (1986). Complementation of sensitivity to alkylating agents in *Escherichia coli* and Chinese hamster ovary cells by expression of a cloned bacterial DNA repair gene. *EMBO J.*, **5**, 3195-3200.

KOHN, K.W. (1977). Interstrand Cross-linking of DNA by 1,3,Bis(2-chloroethyl)1-nitrosourea and other 1-(2-haloethyl)-1-nitrosoureas. *Cancer Res.*, **37**, 1450-1454.

KOSAK, M. (1987). An analysis of 5'-noncloning sequences from 699 vertebrate messenger RNAs. *Nucleic Acids Res.*, **15**, 8125-8148.

KUNKEL, T., ROBERTS, J.D. & ZAKOUR, R.A. (1987). Rapid and efficient site-specific mutagenesis without phenotypic selection. *Methods in Enzymol.*, **154**, 367-382.

MADOX-JONES, H. & MAURO, F. (1975). Site of action of cytotoxic agents in the cell life cycle. In *Antineoplastic and Immunosupressive Agents*. Sartorelli, A. & John, D. (eds) pp. 205-219. *Handbook of Exp. Pharmacol.*, **38**, 205-219. Springer Berlin, Heidelberg: New York.

MANIATIS, T., FRITSCH, E.F. & SAMBROOK, J. (1982). In *Molecular Cloning: A Laboratory Manual*. Cold Spring Harbour Laboratory.

MARGISON, G.P., COOPER, D.P. & BRENNAND, J. (1985). Cloning of the *E. coli* $O^6$-methylguanine and methylphosphotriester methyltransferase gene using a functional DNA repair assay. *Nucleic Acids Res.*, **13**, 1939-1952.

MARGISON, G.P. & O'CONNOR, P.J. (1990). Biological consequences of reactions with DNA: Role of specific lesions. In *Handbook of Exp. Pharm.*, **94**, Grover, C.S. & Grover, P.L. (eds) pp. 547-571.

MORTEN, J.E.N., BAYLEY, L., WATSON, A.J., WARD, T.H., POTTER, P.M., RAFFERTY, J.A. & MARGISON, G.P. (1992). Upregulation of $O^6$-alkylguanine-DNA alkyltransferase expression and the presence of double minute chromosomes in alkylating agent selected Chinese hamster cells. *Carcinogenesis*, **13**, 483-487.

MYRNES, B., NORSTRAND, K., GIERCKSKY, K.-E., SJUNNESKOG, C. & KROKAN, H. (1984). A simplified assay for $O^6$-methylguanine-DNA methyltransferase activity and its application to human neoplastic and non-neoplastic tissues. *Carcinogenesis*, **5**, 1061-1064.

MUSK, S.R.R., HATTON, D.H., BOUFFLER, S.D., MARGISON, G.P. & JOHNSON, R.T. (1989). Molecular mechanisms of alkylation sensitivity in Indian muntjac cell lines. *Carcinogenesis*, **10**, 1299-1306.

OLSSON, M. & LINDAHL, T. (1980). Repair of alkylated DNA in *E. coli*. *J. Biol. Chem.*, **255**, 10569-10571.

POTTER, P.M., WILKINSON, M.C., FITTON, J., CARR, F.J., BRENNAND, J., COOPER, D.P. & MARGISON, G.P. (1987). Characterisation and nucleotide sequence of *ogt*, the $O^6$-alkylguanine-DNA-alkyltransferase gene of *E. coli*. *Nucleic Acids Res.*, **15**, 9177-9193.

ROBINS, P., HARRIS, A.L., GOLDSMITH, I. & LINDAHL, T. (1983). Cross-linking of DNA induced by chloroethylnitrosoureas is prevented by $O^6$-MeG-DNA methyltransferase. *Nucleic Acids Res.*, **11**, 7743-7758.

ROGERS, K.J. & PEGG, A.E. (1977). Formation of $O^6$-methylguanine by alkylation of rat liver, colon and kidney DNA following administration of 1,2-Dimethylhydrazine. *Cancer Res.*, **37**, 4082-4088.

SAFFHILL, R., MARGISON, G.P. & O'CONNOR, P.J. (1985). Mechanisms of carcinogenesis induced by alkylating agents. *Biochim. Biophys. Acta*, **823**, 111-145.

SAMSON, L., DERFLER, B. & WALDSTEIN, E.A. (1986). Suppression of human DNA alkylation defects by *Escherichia coli* DNA-repair genes. *Proc. Natl Acad. Sci. USA*, **83**, 5607-5610.

SEDGWICK, B. (1983). Molecular cloning of a gene which regulates the adaptive response to alkylating agents in *Escherichia coli*. *Mol. Gen. Genet.*, **191**, 466-472.

SILINSKAS, K.C., ZUCKER, P.F. & ARCHER, M.C. (1985). Formation of $O^6$-methylguanine in rat liver DNA by nitrosamines does not predict initiation of preneoplastic foci. *Carcinogenesis*, **6**, 773-775.

WILKINSON, R. & NIAS, A.H.W. (1971). Cell Kinetics and HN2 sensitivities of HeLa sublines with different colony growth rates. *Experimental Cell Res.*, **65**, 73-80.

WILKINSON, M.C., POTTER, P.M., CAWKWELL, L., GEORGIARDIS, P., PATEL, D., SWANN, P.F. & MARGISON, G.P. (1989). Purification of the *E. coli ogt* gene product to homogeneity and it's rate of action on $O^6$-Methylguanine, $O^6$-Ethylguanine and $O^4$-Methylthymine in dodecadeoxyribonucleotides. *Nucleic Acids Res.*, **17**, 8475-8484.

WU, Z., CHAN, C.-L., EASTMAN, A. & BRESNICK, E. (1992). Expression of human $O^6$-methylguanine-DNA methyltransferase in a DNA excision repair-deficient Chinese hamster ovary cell line and its response to certain alkylating agents. *Cancer Res.*, **52**, 32-35.